# Gout Initially Mimicking Rheumatoid Arthritis and Later Cervical Spine Involvement

**DOI:** 10.1155/2014/357826

**Published:** 2014-12-09

**Authors:** Eduardo Araújo Santana Nunes, Adroaldo Guimarães Rosseti, Daniel Sá Ribeiro, Mittermayer Santiago

**Affiliations:** ^1^Department of Rheumatology, Santa Izabel Hospital, Praça Almeida Couto 500, 40050-410 Salvador, BA, Brazil; ^2^Department of Neurosurgery, Santa Izabel Hospital, Praça Almeida Couto 500, 40050-410 Salvador, BA, Brazil; ^3^Department of Radiology and Image Memorial Radiology Service, Santa Izabel Hospital, Praça Almeida Couto 500, 40050-410 Salvador, BA, Brazil; ^4^Serviços Especializados em Reumatologia da Bahia, Rua Conde Filho 117, Graça, 40150-150 Salvador, BA, Brazil

## Abstract

Gout is clinically characterized by episodes of monoarthritis, but if not treated properly, it can lead to a chronic polyarthritis, which may eventually mimic rheumatoid arthritis (RA). We present the case of a 59-year-old man, with a history of symmetrical polyarthritis of the large and small joints with later development of subcutaneous nodules, which was initially misdiagnosed as RA, being treated with prednisone and methotrexate for a long period of time. He complained of occipital pain and paresthesia in his left upper limb, and computed tomography (CT) and magnetic resonance imaging (MRI) revealed the presence of an expansive formation in the cervical spine with compression of the medulla. He was admitted for spinal decompressive surgery and the biopsy specimen demonstrated a gouty tophus. Chronic gout can mimic RA and rarely involves the axial skeleton, and thus its correct diagnosis and the implementation of adequate therapy can halt the development of such damaging complications.

## 1. Introduction

Gout is a common disturbance of purine metabolism, in which the deposition of sodium monourate crystals in the synovial tissue produces acute arthritis of the peripheral joints. Localized urate deposits may also be found in extra-articular sites, or tophi, which in general are present in the later stages of the disease that is commonly found in hands, feet, bursa of the olecranon, and helix of the ear.

Involvement of the axial skeleton is a rare complication of gouty arthritis. We describe a patient with a deforming polyarticular and symmetrical arthritis, with subcutaneous nodules resembling rheumatoid arthritis (RA) that later on developed a severe cervicobrachialgia secondary to a tophaceous deposit in the cervical spine.

## 2. Case Report

**Figure 1:**
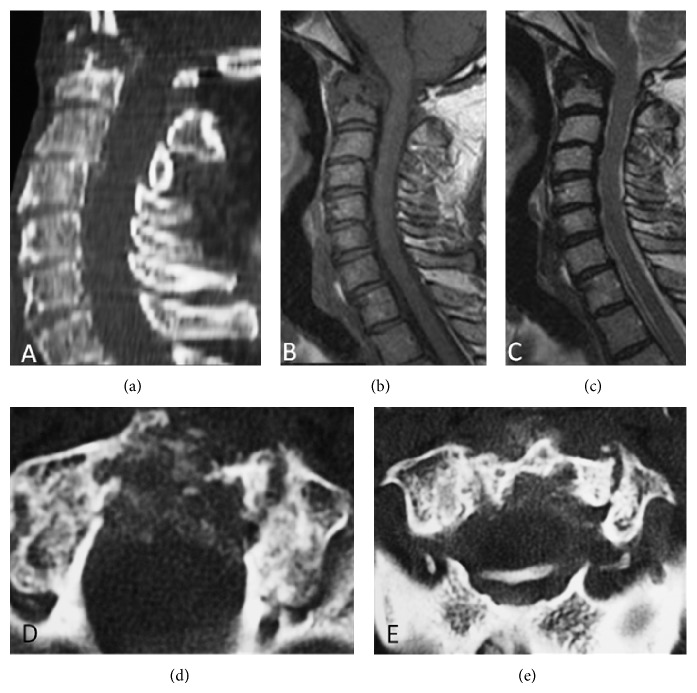
(a) Sagital computed tomography (CT) image shows a destructive lesion of odontoid process. (b) Sagital T1-weighted spin eco and (c) sagital T2-weighted spin eco demonstrating a hypointense mass with compression of the spinal cord. (d) and (e) Axial CT images of this region with well-defined calcification foci within the mass suggesting microcrystal deposition.

**Figure 2:**
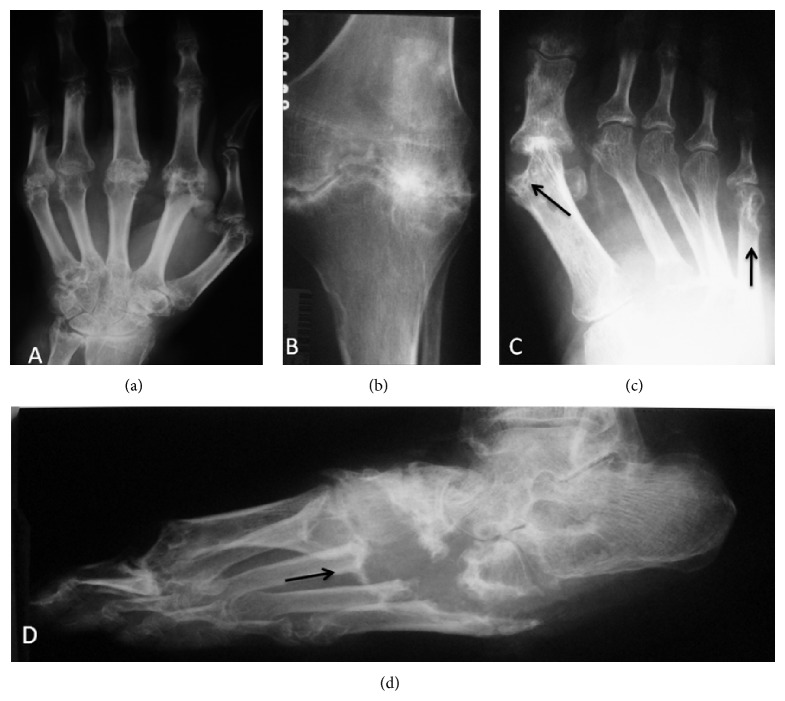
Radiographs of hand (a), knee (b), foot (c), and ankle (d) showing severe destructive changes secondary to tophaceous gout with erosion, soft tissue swelling (tophi), articular space narrowing, and proliferative bone abnormalities. Note the well-defined extra-articular large erosions with overhanging margin and surrounding bone eburnation (arrows). The uniform joint space narrowing with normal bone density in knee and wrist is typical of late gout disease.

The patient, a 59-year-old Brazilian black man, had been previously diagnosed with RA based on the history of polyarthritis of the large and small joints, morning stiffness, subcutaneous nodules, and joint deformities in his hands and feet. He was taking methotrexate (10 mg/week) and prednisone (5 mg/day) which partially controlled his symptoms. He also suffered from systemic arterial hypertension, which got controlled with captopril (100 mg/day), propranolol (80 mg/day), and hydrochlorothiazide (25 mg/day). Over the last few months the patient experienced severe headaches in the occipital region and cervicalgia with irradiation to the left upper limb, resembling the possibility of atlantoaxial subluxation. On physical examination, it was found that his general condition was good, with a blood pressure level of 130/80 mm Hg; he had “swan-neck” deformity of the fingers, “camel back” deformities in his wrists, and fixed deformities in his feet and knees with a significant limitation of movement amplitude both passively and actively. There were several subcutaneous nodules in extension surfaces of his joints, mainly in elbows. There were left brachial paresis graded 4/5, hypertrophy of the left shoulder muscles, and hyperreflexia graded 3/4 in the upper limbs; however, proprioceptive, vibratory, and pain sensitivity were preserved and there were signs of autonomic dysfunction. Cardiovascular and respiratory examinations were unremarkable. The laboratory investigation showed hyperuricemia (8.6 mg/dL), hypertriglyceridemia, and elevation in creatinine values (1.5 mg/dL). The rheumatoid factor (RF) and anti-CCP antibodies were negative. Cervical magnetic resonance imaging (MRI) showed destructive lesion of the odontoid process and computed tomography (CT) revealed part of C1 vertebra with hyperdense foci suggesting microcrystal deposition (Figure 1). Radiographs of the knee, feet, ankle, and hands showed periarticular soft tissue swelling with extensive bone erosions and joint space narrowing with normal bone density (Figure 2). Based on these findings, the diagnosis of tophaceous and mutilans-type gout was established and the use of colchicine and allopurinol was initiated.

The patient was admitted for two neurosurgical procedures: initially occipitocervical arthrodesis and later exeresis of the mass in the odontoid process. The histopathological study demonstrated an accumulation of an acellular amorphous substance with crystals and fibrosis and a granulomatous reaction foreign body type involving the soft parts and bone tissue and the absence of neoplastic cells.

During subsequent evaluations in the outpatient clinic, the patient no longer complained of cervical pain and showed improvement of the left brachial paresis.

## 3. Discussion

Gout is one of the most ancient clinical entities with evidence of its description dating back to times preceding the descriptions made by Hippocrates [1]. It primarily affects the distal joints of the appendicular skeleton, initially with a monoarticular pattern of involvement and later, particularly when not well treated, becomes polyarticular. It can eventually mimic RA, as in the present case, which was initially treated erroneously as this condition.

There are other descriptions of gout mimicking RA in the literature [2] as well as rare cases of coexisting gout and RA [3]. The latter was not the case with our patient as the bone erosions observed in the radiographs were typical of gout arthropathy, and the RF and anti-CCP antibodies were negative.

Involvement of the axial skeleton by gouty tophi has been described in the literature in around 100 cases [4] and may be associated with compression of the spinal cord. However, a recently published paper revealed the presence of spinal gout, as identified by CT, in 12 out of 42 (29%) gouty patients, mainly in lumbar and none in cervical spine and there was no association between symptoms and axial gout [5]. Patients may present with a variety of symptoms, such as isolated lumbar or cervical pain or various other neurological syndromes, depending on the level of tophi deposition in the spinal cord. A history of peripheral gout for several months or years preceding the neurological manifestations occurs in the large majority of patients and helps in the clinical diagnosis [6]. Nevertheless, it is worth pointing out that, in the literature, there are reports of gout with axial involvement with nerve compression without a previous history of peripheral arthritis [7]. The concomitant presence of “spinal gout” and the peripheral joint involvement with a rheumatoid-like pattern as seen in our patient is unique. In theory, the spinal complication could probably have been avoided if the diagnosis of gout had not been delayed by the articular presentation suggestive of RA, and the tophi confounded with rheumatoid nodules and the treatment for gout had been promptly established.

In axial gout, any segment of the spine and its components (vertebral bodies, pedicles, lamina, ligaments, interapophyseal cartilage, and epidural and intradural spaces) may be involved, with lumbar involvement being the most common [8]. The mechanism of compression differs from level to level: (a) at the cervical level, the symptoms of pain and instability are generated by the paravertebral involvement and consequent ligamentous instability; (b) at the thoracic level, it deposits in the extramural space with medullar compression; (c) at the lumbar level, the symptoms result from the radicular compression; (d) at the sacroiliac level, it deposits in the hyaline cartilage of the interarticular space and adjacent bones causing the symptoms [9].

Because of the rarity of axial involvement in gout, before making a diagnosis, it is necessary to exclude other possible etiologies such as spondylodiscitis, infection, and neoplasia [10]. For this purpose, it is necessary to use techniques such as MRI and CT, since plain radiographs are generally negative or may show only degenerative alterations and bone erosions [6, 8–10]. CT can show facet joint erosions that are more consistent with gout than with degenerative alterations [11]. MRI can be very useful in a differential diagnosis since the tophi present a low signal in the pondered sequence in T1 and a low or high heterogeneous signal in the pondered images in T2 (the high signal is owing to the filling of the amorphous center of the tophus with high protein content [7] and the heterogeneity and to the varied levels of calcium deposits in the tophus [12]) and present variable peripheral enhancement after gadolinium administration.

In conclusion, chronic gout can mimic RA and rarely involves the axial skeleton, and thus its correct diagnosis and the implementation of adequate therapy can halt the development of such damaging complications.
